# The Use of Dixon Magnetic Resonance Imaging Methods for the Quantification of Rotator Cuff Fatty Infiltration: A Systematic Review

**DOI:** 10.3390/muscles3020013

**Published:** 2024-05-19

**Authors:** Andrew J. Nasr, Joshua Harris, Jijia Wang, Michael Khazzam, Nitin B. Jain, Yi-Ting Tzen, Yen-Sheng Lin

**Affiliations:** 1 Department of Applied Clinical Research, University of Texas Southwestern, Dallas, TX 75390, USA; 2 School of Medicine, Northeast Ohio Medical University, Rootstown, OH 44272, USA; 3 Department of Orthopaedic Surgery, University of Texas Southwestern, Dallas, TX 75390, USA; 4 Department of Physical Medicine and Rehabilitation, University of Michigan, Ann Arbor, MI 48108, USA; 5 Department of Physical Medicine and Rehabilitation, University of Texas Southwestern, Dallas, TX 75390, USA

**Keywords:** Dixon MRI, rotator cuff, shoulder, fatty infiltration, atrophy, muscle degeneration

## Abstract

Fatty infiltration of the rotator cuff muscles is very common following rotator cuff tears and is one of the most important factors in determining treatment. Current clinical practice relies on subjective evaluation of fatty infiltration through categorical scoring based on the Goutallier classification system. The Dixon magnetic resonance imaging (MRI) sequence provides flexibility in selecting echo times for water–fat separation. The Dixon method, therefore, has the potential to provide robust and high-quality fat quantification that allows for more accurate calculation of fat fraction (%Fat) of the rotator cuff muscles than the Goutallier classification system. However, significant variance exists in sequencing and post-processing methodology within the recent application of Dixon sequences to quantify rotator cuff fatty infiltration. In this paper, we conducted a systematic review to synthesize the relevant literature utilizing Dixon sequencing for the quantification of rotator cuff fatty infiltration. The literature search was extracted from 1094 articles, with 12 studies included in the final review. Regardless of the varying sequencing pattern and post-processing techniques among studies, the findings suggest the Dixon method is reliable for quantitatively calculating the fat fraction of the rotator cuff muscles, even at very low levels of fatty infiltration. In addition, a quantitative difference in fat fraction was observed between participants with different degrees of tear vs. those without any shoulder pathologies. Multi-point Dixon imaging has the potential to be utilized clinically to objectively quantify fatty infiltration and may lead to improved clinical decision making for patients with rotator cuff tears.

## 1. Introduction

Rotator cuff disease is a highly prevalent musculoskeletal condition with more than 250,000 rotator cuff repairs performed annually in the United States [1,2,3]. In untreated rotator cuff tears, progressive and irreversible fatty infiltration and atrophy occur over time [4]. Fatty infiltration is influenced by the size of the tear and the number of involved tendons and leads to a loss of muscle strength and function, and a higher degree of fatty infiltration is associated with poor surgical and non-surgical outcomes [5]. The natural history of fatty infiltration remains poorly understood; however, animal models show fatty infiltration and muscle atrophy progress steadily during the initial four months after tendon detachment [6]. Additionally, patients with a higher degree of fatty infiltration are often considered poor surgical candidates due to concerns over tendon healing. Despite the clinical importance of fatty infiltration, current clinical standards to evaluate fatty infiltration are based on the semi-quantitative 5-point ordinal Goutallier classification system (Grade 0: normal muscle; Grade 1: some fatty streaks; Grade 2: more muscle than fat; Grade 3: equal amounts of fat and muscle; Grade 4: more fat than muscle) [7,8]. This is further exacerbated by the growing obesity epidemic in the United States as obesity is shown to influence fatty infiltration of the rotator cuff muscles [9]. Given the clinical and prognostic significance of rotator cuff fatty infiltration, a robust and objective quantitative measure of fatty infiltration should be the clinical gold standard.

**Figure 1:**
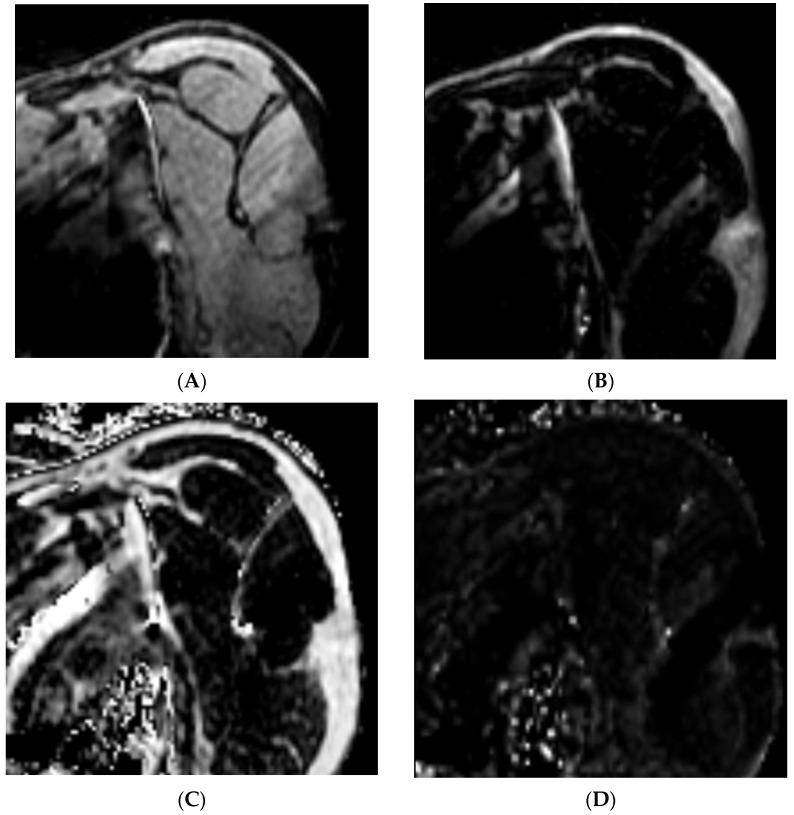
Dixon sequencing generates four unique data sets of the same MR image: (**A**) water only, (**B**) fat only, (**C**) in phase, and (**D**) opposed phase.

The Dixon method of magnetic resonance imaging (MRI) is a chemical shift-based water–fat technique where data are collected at different echo times allowing for the separation of water and fat by simple summation and subtraction of the images [10]. The water–fat separation technique generates four unique image sets of the same acquisition. Figure 1 demonstrates the four unique data sets of the same MR image collected on an individual with rotator cuff tear: water-only (1A), fat-only (1B), in-phase (1C), and opposed-phase images (1D). This separation allows for the quantification of intramuscular fat by comparing the signal intensities of water and fat in these images [11]. Thus, the water and fat separation allows for accurate fat quantification across many different pulse sequences and clinical applications. The data can then be combined to calculate fat fraction using the following formula [12,13,14,15]:

Fat Fraction = [S_Fat_/(S_Water_ + S_Fat_)] × 100% (1)

where S is the signal intensity of the corresponding water- or fat-only image. Further refinement of the Dixon method has led to the development of additional sequencing with flexible echo times and in the case of the modified Dixon method, the ability to select the shortest echo time to achieve a high-quality image, subsequently reducing the scanning time for patients [16]. The use of the Dixon method in musculoskeletal imaging has increased in popularity with the availability of standardized spin-echo sequences [17,18]. The value of the Dixon method is of particular interest in the management of patients with rotator cuff tears, as fatty infiltration is a prevalent complication and a strong prognostic factor, particularly with current clinical standards relying on a more qualitative classification method [5]. Therefore, the purpose of this systematic review is to synthesize the existing literature with reference to imaging and post-processing methodologies for the quantification of rotator cuff fatty infiltration. The primary aim is to systematically summarize the application of the Dixon method to quantitatively measure fatty infiltration of the rotator cuff muscles. The secondary aim is to evaluate the reliability and accuracy of the Dixon method and its potential future use as a clinical tool in surgical and non-surgical planning.

## 2. Results

### 2.1. Literature Search Results

**Figure 2:**
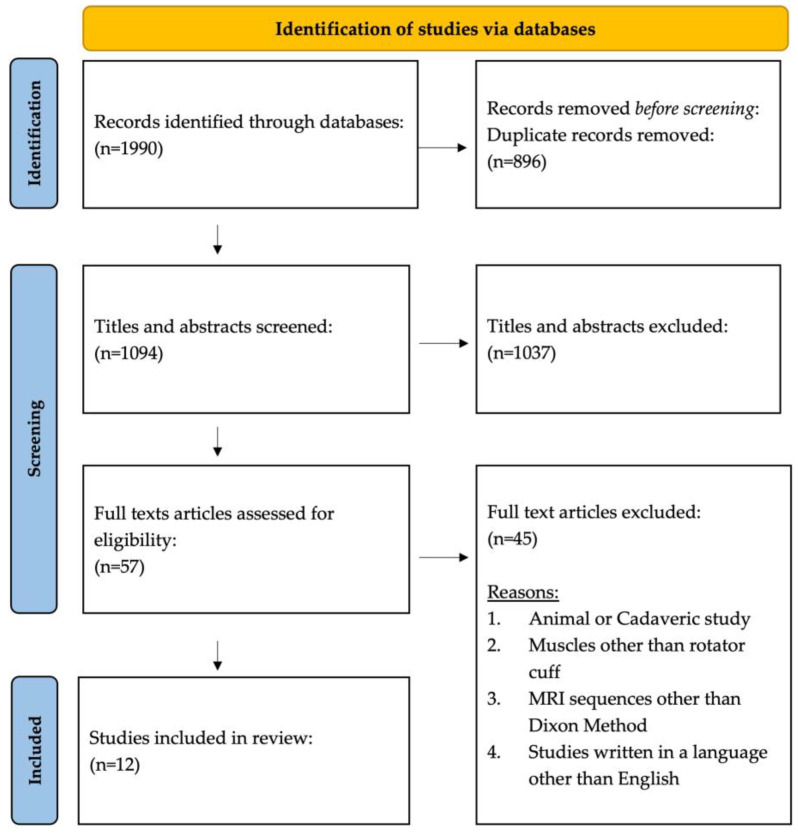
PRISMA flow diagram and article selection process.

A flowchart diagram of the study selection process in accordance with the Preferred Reporting Items for Systematic Reviews and Meta-Analyses (PRISMA) is shown in Figure 2. The search identified 1990 published studies and 896 articles were removed due to duplication. In total, 1037 articles were then excluded based on the title and abstract reviews. Following 57 eligible articles being examined through full-text reviews, a total of 12 studies that met inclusion and exclusion criteria were included. The criteria are detailed in Figure 2. In total, six prospective studies, four retrospective studies, one case–control, and one case series were included. Of the 12 articles included in the review, 6 aimed to quantify the fat fraction of the rotator cuff and the associated shoulder musculature, 3 assessed the reliability of Dixon sequencing, 2 evaluated the correlation of Dixon sequencing to a reference test, and 1 investigated the diagnostic utility of Dixon sequencing compared to a reference test.

### 2.2. Participant Characteristics

A total of 1054 participants were included across the twelve studies with sample sizes ranging from 13 to 359 and 51% being female. The majority of participants had complaints of shoulder pain (91.8%) with those remaining serving as healthy controls (8.2%). Participant characteristics are listed in Table 1.

### 2.3. Characteristics of MR Imaging Post-Processing

A summary of imaging and post-processing protocols can be found in Table 1. Post-processing of the raw Dixon images varied across the studies with half utilizing a separate dedicated software for quantification of fatty infiltration and the other half utilizing the institution’s Picture Archiving and Communication System (PACS).

### 2.4. Characteristics of MR Imaging Sequences

Imaging parameters and protocols of the three studies utilizing T2*-corrected Dixon imaging were summarized in Table 2 and the nine studies utilizing multiple-point Dixon imaging were summarized in Table 3. Eleven of the twelve included studies were completed with a 3.0 Tesla MRI and all twelve utilized a dedicated shoulder coil. For all included studies, six studies used two echo times, two studies used three echo times, and four studies used six echo times.

**Table 1:** Summary of 12 included articles.

Study	Study Design, Level of Evidence	N (Male/Female)	MRI Unit	Fat Fraction Software	Dixon Images Used
Lee 2015 [19]	Retrospective, III	89 (42/47)	3T Siemens	PACS	In Phase and Opposed Phase
Nozaki 2015 [20]	Prospective, II	359 (185/174)	3T Siemens	PACS	Fat and Water Only
Agten 2016 [21]	Prospective Case–Control, III	60 (41/19)	1.5T Siemens	MATLAB	Fat and Water Only
Nozaki 2016 [22]	Prospective, II	50 (18/32)	3T Siemens	PACS	Fat and Water Only
Horiuchi 2017 [23]	Retrospective, III	200 (86/114)	3T Siemens	PACS	In Phase and Fat Only
Matsumura 2017 [12]	Prospective, II	40 (20/20)	3T GE	OsiriX MD	Fat and Water Only
Kalin 2018 [13]	Prospective, II	76 (37/39)	3T Philips	Myrian	Fat and Water Only
Yoon 2018 [24]	Prospective, II	24 (10/14)	3T Siemens	PACS	Fat and Water Only
Khanna 2019 [14]	Retrospective, III	13 (5/8)	3T Siemens	AnalyzeDirect and MATLAB	Fat and Water Only
Kalin 2019 [25]	Prospective, II	40 (28/12)	3T Siemens	Syngo.Via	In Phase/Opposed Phase and Fat and Water Only
Hahn 2021 [15]	Retrospective, III	57 (30/27)	3T Philips	PACS	Fat and Water Only
Xu 2023 [26]	Prospective Case Series, IV	46 (15/31)	3T Siemens	Syngo.Via (VB10B)	Fat and Water Only

PACS, picture archiving and communication system; T, Tesla.

**Table 2:** Studies reporting on the measurement of the rotator cuff muscle fat content with T2*-corrected Dixon imaging.

Study	N (Male/Female)	Dixon Sequencing	Dixon Parameters	T2* Correction	Fat Fraction Calculation
Lee 2015 [19]	89 (42/47)	3D 3-point Dixon VIBE	TR (ms) = 12.5 TE1, TE2, TE3 (ms) = 2.45, 6.12, 9.8 Thickness (mm) = 3.0 Flip Angle (^o^) = 11 Matrix (pixel) = 256 × 256 FOV (mm) = 140 × 140 TA (min:s) = 1:09	Using a linear fit in log space, T2* value per pixel was estimated from two in-phase echoes (TE 2.45, 9.8) and used to correct the signal intensity of the opposed-phase (TE 6.12) and the first in-phase (TE 2.45) echoes.	[S_Fat_/(S_Fat_ + S_Water_)] × 100
Agten 2016 [21]	60 (41/19)	3D Multi-echo Dixon	TR (ms) = 20 TE1–TE6 (ms) = 2.39, 4.78, 6.34, 7.90, 9.46, and 11.02 Thickness (mm) = 3.0 Flip Angle (^o^) = 10 Matrix (pixel) = 144 × 192 FOV (mm) = 120 × 160 TA (min:s) = 3:29	Multi-peak fat spectral model developed for liver fat by Zhong [27].	Algorithm automatically calculated a fat percentage map
Yoon 2018 [24]	50 (18/32)	3D Multi-point Dixon VIBE	TR (ms) = 9.15 TE1–TE6 (ms) = 1.09, 2.46, 3.69, 4.92, 6.15, and 7.38 Thickness (mm) = 4.0 Flip Angle (^o^) = 4 Matrix (pixel) = 120 × 101 FOV (mm) = 300 × 300 TA (min:s) = 1:20	T2*-corrected images acquired in the oblique sagittal plane.	The algorithm automatically calculated a fat percentage map based on three regions of interest.

TR, repetition time; TE, echo time; FOV, field of view; TA, acquisition time; VIBE, volumetric interpolated breath-hold examination.

**Table 3:** Studies reporting on the measurement of the rotator cuff muscle fat content with multiple-point Dixon imaging.

Study	N (Male/Female)	Dixon Sequencing	Dixon Parameters	Fat Fraction Calculation
Nozaki 2015 [20]	359 (185/174)	3D 2-point Dixon VIBE	TR (ms) = 6.5 TE1 & TE2 (ms) = 1.225 & 2.4 Thickness (mm) = 2.5 Flip Angle (^o^) = 10 Matrix (pixel) = 128 × 128 FOV (mm) = 196 TA (min:s) = 2:30	S_Fat_/(S_water_ + S_Fat_) = S_Fat_/S_In_
Nozaki 2016 [22]	50 (18/32)	3D 2-point Dixon VIBE	TR (ms) = 6.5 TE1 & TE2 (ms) = 1.225 & 2.4 Thickness (mm) = 2.5 Flip Angle (^o^) = 10 Matrix (pixel) = 128 × 128 FOV (mm) = 196 TA (min:s) = 2:30	S_Fat_/(S_water_ + S_Fat_) = S_Fat_/S_In_
Horiuchi 2017 [23]	200 (86/114)	3D 2-point Dixon VIBE	TR (ms) = 6.5 TE1 & TE2 (ms) = 1.225 & 2.4 Thickness (mm) = 2.5 Flip Angle (^o^) = 10 Matrix (pixel) = 128 ×128 FOV (mm) = 196 TA (min:s) = 2:30	S_Fat_/(S_Water_ + S_Fat_) = S_Fat_/S_In_
Matsumura 2017 [12]	40 (20/20)	3D 2-point Dixon	TR (ms) = 4.2 TE (ms) = 1.7 Thickness (mm) = 2.0 Flip Angle (^o^) = 12 Matrix (pixel) = 288 × 224 FOV (mm) = 260 TA (min:s) = 2:20	S_Fat_/(S_Water_ + S_Fat_)
Kalin 2018 [13]	76 (37/39)	3-point Dixon	TR (ms) = 9.4 TE1–TE3 (ms) = 3.6, 5.3, 7.0 Thickness (mm) = unspecified Flip Angle (^o^) = 10 Matrix (pixel) = 744 FOV (mm) = 560 × 300 TA (min:s) = 5:39	S_Fat_/(S_Water_ + S_Fat_)
Khanna 2019 [14]	13 (5/8)	3D Multi-echo 2-point Dixon	TR (ms) = 9.4 TE1 & TE2 (ms) = 1.29 & 2.52 Thickness (mm) = 2.0 Flip Angle (^o^) = 9 Matrix (pixel) = 320 × 320 FOV (mm) = 380 TA (min:s) = unspecified	S_Fat_/(S_Water_ + S_Fat_) × 100
Kalin 2019 [25]	40 (28/12)	3D Multi-echo Dixon	TR (ms) = 13.22 TE1–TE6 (ms) = 1.07, 3.14, 5.21, 7.28, 9.35, 11.42 Thickness (mm) = 2.5 Flip Angle (^o^) = 5 Matrix (pixel) = 160 × 160 FOV (mm) = 200 TA (min:s) = unspecified	Not specified
Hahn 2021 [15]	57 (30/27)	T1W mDixon TSE MRA	TR (ms) = 498–608 TE1–TE6 (ms) = 10–30 and automatically calculated shorted TE Thickness (mm) = 3.0 Flip Angle (^o^) = unspecified Matrix (pixel) = 320 × 256 FOV (mm) = 140 TA (min:s) = 4:20–25	S_Fat_/(S_Water_ + S_Fat_) × 100
Xu 2023 [26]	46 (15/31)	6-point Dixon	TR (ms) = 4.32 TE (ms) = 1.35, 2.58 Thickness (mm) = 2.0 Flip Angle (^o^) = 9 Matrix (pixel) = 288 × 320, 227 × 320 FOV (mm) = 346 × 346 TA (min:s) = 2:36, 2:35	S_Fat_/(S_Water_ + S_Fat_) × 100

TR, repetition time; TE, echo time; FOV, field of view; TA, acquisition time; VIBE, volumetric interpolated breath-hold examination; TSE, turbo spine echo; MRA, magnetic resonance arthrogram; S_Fat_, signal intensity of fat-only image; S_Water_, signal intensity of water only image; S_In_, signal intensity of in-phase image.

### 2.5. Assessment of Methodological Quality

A summary of the Grading of Recommendations Assessment, Development, and Evaluation (GRADE) assessment of methodological quality and risk of bias and applicability concerns are presented in Table 4 and Table 5, respectively. This framework encompasses six key domains: risk of bias, imprecision, inconsistency, indirectness, publication bias, and magnitude of effect size. Evidence quality may diminish due to factors such as study limitations, inconsistency, indirectness, imprecision, and publication bias. Overall, 6 of the 12 articles had low to no risk of bias based on the Quality Assessment of Diagnostic Accuracy Studies (QUADAS-2) [28]. For the overall quality of grading for the 12 included studies, we adapted the criteria and utilized five domains of the GRADE framework due to inconsistent or unavailable reports on effect size (Table 4) [29]. Three studies were graded 5, seven studies were graded 4, and two studies were graded 3.

### 2.6. Summary of Results

#### 2.6.1. Quantification of Fat Fraction

A summary of the study results can be found in Table 6. In total, 6 of the 12 articles quantify the fat fraction of at least one rotator cuff muscle; however, the sample populations varied from completely asymptomatic to confirmed full-thickness tears. The supraspinatus fat fraction ranged between 7.6 and 15.1% in participants without any shoulder pathology [12,13,20,25], 2.5% in patients with adhesive capsulitis [25], and 16.6–44.0% in patients with rotator cuff tears [12,20]. Among patients with a rotator cuff tear, the supraspinatus fat fraction is the highest in people with a confirmed full tear (0.285 ± 0.123), followed by those with a partial tear (0.166 ± 0.067) [20]. Another study assessing fat fraction values and muscle volume of the whole 3D rotator cuff muscles showed similar results that patients with supraspinatus tear only had higher supraspinatus fat fraction as compared to participants without tear (27.7 ± 11.4% vs. 15.1 ± 2.4%) [12]. In addition, they also showed that patients with massive tears had significantly higher fat fraction in both the supraspinatus (44.0 ± 9.9%, *p* < 0.001) and infraspinatus (41.2 ± 9.8%, *p* < 0.001). This increase in fat fraction is concurrent with a decrease in lean muscle volume in the supraspinatus and infraspinatus and an increase in lean muscle volume in the teres minor [12].

In terms of utilizing pre-operative fat fraction as a predictor of surgery outcome, Nozaki and colleagues showed that pre-operative fat fraction values of supraspinatus and subscapularis were significantly higher in the failed repair group (37.0% vs. 19.5% and 19.7% vs. 12.3%, respectively, *p* < 0.001). They also examined the post-operative fat fraction of the subscapularis at one-year follow-up and found that the fat fraction was significantly higher in the intact repair versus the normal tendon group (21.3% vs. 12.2%, *p* < 0.001); however, there were no significant differences between failed repair and intact repair groups [22]. Kalin and colleagues provided mean values for fat fraction and muscle volume of the rotator cuff muscles as well as the deltoid muscles in asymptomatic male and female adults. Interestingly, women had higher subscapularis fat fraction values than men (7.5 ± 2.8% vs. 6.8 ± 1.9%, *p* = 0.001) but not in any other muscle [13]. In a follow-up study in 2019, Kalin and colleagues established mean values of fat fraction in an asymptomatic population and established age-associated changes between genders. The authors found a significant positive correlation between age and rotator cuff muscle fat fraction values (*r* = 0.273, *p* < 0.05) [25]. Yoon and colleagues described fat fraction values in patients with adhesive capsulitis who developed fatty infiltration. Only the supraspinatus had a significant increase in fat fraction with positive signs of extracapsular hyperintensity, such as high signal intensity outside of the axillary recess capsule on the oblique coronal T2-weighted fat-suppressed images (3.00 ± 1.74% versus 1.81 ± 0.80%, *p* = 0.022) [24]. Of the six studies investigating fat fraction values, only three reported sex differences [13,24,25]. Dixon sequencing and post-processing protocols varied across all six studies with heterogeneous participant characteristics and various study designs, limiting the ability to pool raw data for further meta-analysis. 

#### 2.6.2. Dixon Sequencing Reliability and Accuracy

In total, 3 of the 12 studies reported reliability and agreement measures. The inter-rater reliability ranges between 0.82 and 0.99 from three studies [14,21,23]. Agten and the colleagues assessed the reliability of multi-echo 3D Dixon sequencing to quantify low levels of fat content in the supraspinatus with good to excellent test–retest reliability [rater 1 ICC = 0.757, (95% confidence interval (CI) 0.461–0.902, *p* < 0.0005) and rater 2 ICC = 0.873 (95% CI 0.696–0.950, *p* < 0.0005)]. Inter-rater reliabilities for a small region of interests [ICC = 0.893, (95% CI 0.825–0.935 *p* < 0.0005)] and a large region of interests [ICC = 0.967, (95% CI 0.944–0.981, *p* < 0.0005)] were excellent [21]. Horiuchi and colleagues also evaluated the reliability of a 2-point Dixon sequence among five independent raters with excellent inter-observer agreement [ICC = 0.893, (95% CI 0.845–0.931)] and excellent intra-rater coefficients for all raters [ICCs > 0.893 (95% CI 0.819–0.975)] [23]. Khanna and colleagues aimed to establish reproducibility and the minimal clinically important differences (MCID) to define 3D whole muscle volume, as well as the distribution of fatty infiltration using a 3D Multi-echo Two-point Dixon. Intra-rater reliability of sagittal and axial divisions of the 3D rotator cuff muscles were very good to excellent [ICC = 0.93–0.99 (95% CI 0.79–0.99) and ICC = 0.78–0.99 (95% CI 0.28–0.99)], respectively. Inter-rater reliability for fat fraction values [ICC = 0.82–0.99 (95% CI 0.22–0.99)] and volume [ICC = 0.92–0.99 (95% CI 0.75–0.99)] were very good to excellent, respectively [14].

Lee and colleagues assessed the usefulness of a T2*-corrected 3-point Dixon sequence to quantify fat fraction validated against a phantom study and found excellent agreement (*r* = 0.9096, *p* < 0.01). T2*-corrected maps produced higher quality fat fraction maps than uncorrected T2* maps in addition to being more strongly correlated with the phantom study [19]. Xu and colleagues developed a novel means to evaluate overall fatty infiltration based on predictive modeling by dividing the muscles into sectional accumulation units (SAU). The middle third of the supraspinatus provided the highest correlation coefficients of overall fatty infiltration (Pearson and Spearman correlations of 0.95 and 0.98, respectively, *p* < 0.001) [26]. Hahn and colleagues investigated the diagnostic utility of a modified Dixon sequence in patients with a suspected rotator cuff tear. They found excellent agreement in the pre-operative diagnosis compared to magnetic resonance arthrogram (176 of 183 tendons, 96.2%). Similar accuracy was found in the post-operative evaluation with 55 of 57 tendons showing agreement (96.5%). They also found a strong positive linear correlation with fat fraction values calculated from the Dixon sequence with that of a fat–water phantom (*r* = 0.994, 95% CI 0.731–0.999) [15].

## 3. Discussion

MRI has proven to be an effective tool in the diagnosis of intramuscular fatty infiltration [30]. The use of the Dixon method in quantifying muscular fat content has seen growing interest with the increased availability of spin-echo sequences, a standard in musculoskeletal imaging protocols. The Dixon method has long been available but recent advances in MRI technologies have increased the utility of sequences [17,18]. The utility of the Dixon method is of particular interest in the management of patients with rotator cuff tears with the presentation of fatty infiltration. Using the Dixon-based water–fat separation technique allows for an objective quantitative assessment of the fatty infiltration of the rotator cuff muscles that are not hindered by the subjective and inter-observer variations of the Goutallier classification system [31].

Despite the increasing popularity of the Dixon method, wide variance exists in both the MRI sequencing and post-processing of the images to calculate fat fraction. This systematic review aimed to provide a synthesized assessment of the current state of the literature investigating the application of the Dixon method to quantitatively measure rotator cuff fatty infiltration. To our knowledge, this is the first systematic review to synthesize the use of quantitative Dixon-based MRI applications to characterize rotator cuff fatty infiltration.

Across the twelve studies, only three had overlapping MRI parameters with these studies having similar authorship [20,22,23]. Nine studies implemented a 3D Dixon sequencing utilizing between two and six different echo times (range of 1.07–11.42 ms). The remaining three studies had echo times ranging from 1.35 to 38 ms with Hahn and the colleagues utilizing a sequence to automatically calculate the shortest echo time to produce a quality image. Slice thickness and flip angle were consistent across all twelve studies ranging from 2.0 to 3.0 mm and 5 to 10°, respectively. It is reasonable to conclude that MRI chemical shift imaging is a reliable modality for quantitatively assessing rotator cuff fatty infiltration and accurately determining the percentage of fat in the rotator cuff muscle. A myriad of challenges exists when advocating for changes in clinical practice with additional time and cost commitments posing significant hurdles. One touted benefit of the Dixon method is the short acquisition time [10]. The acquisition times of the twelve articles ranged from 1:29 to 5:29 (minutes/seconds) with the majority being 2:36 or less. There was an almost equal number of men and women included across the twelve studies, 49% and 51%, respectively, with the age of participants ranging from 20 to 91 years. Race and ethnicity were not specified in any of the studies included. As increased age, time from diagnosis, tear size, and location tears are risk factors for the progressive increase in fatty infiltration, future research is warranted to develop the risk stratification tool to streamline the treatment strategies to optimize patient outcomes. A growing body of evidence has shown quantitative MRI, Dixon sequencing specifically, has high accuracy in quantitatively analyzing hepatic fat compared to the gold standard biopsy [32,33]. The results of our systematic review show similar reliability of Dixon MRI to quantitatively measure rotator cuff fatty infiltration. Inter-rater reliability for calculating fat fraction ranged from 0.75 to 0.992 and intra-rater reliability ranged from 0.757 to 0.996.

Recent studies have also shown that magnetic resonance spectroscopy (MRS), ultrasound elastography, and computed tomography (CT) can be useful in measuring the fat fraction of the rotator cuff muscles [29,34,35,36,37]. Proton MR Spectroscopy (^1^H-MRS) is another chemical-shift-based method that has long been used for the determination of the intramyocellular lipids, mostly in live disease [38]. Given the lack of systematic studies involving the use of ^1^H-MRS, Wang and colleagues aimed to evaluate the accuracy, repeatability, and reproducibility of ^1^H-MRS to measure the fat fraction of the rotator cuff muscles [39]. This study’s findings revealed a high correlation between pooled multi-echo Dixon MRI measures of fat fraction and 1H-MRS measures (R^2^ = 0.749), which was better than the moderate correlation (R^2^ = 0.530) found by Agten et al. [21]. Wang and colleagues highlighted the major advantage of mDixon sequences is that the ROI can take on any shape and size, and can be placed on any image slice for post-image processing, making mDixon more practical for clinical use. Creatine was the important indicator in ^1^H-MRS as the targeted and reference metabolite. However, the individual spectra showed greater deviations than mDixon, posing a difficult clinical assessment of the lipid content [40]. Technical improvement or optimization of the shoulder-specific coil will enhance the assertiveness to measure the fatty infiltration within the targeted rotator cuff muscles. The use of ultrasound elastography is relatively new to the orthopedic literature but does show promise in evaluating muscle quality, including fat fraction [29]. One notable limitation of elastography includes an assumption that skeletal muscle is homogeneous and isotropic. However, new techniques and technology have emerged potentially eliminating this limitation, though more research is needed [41,42]. CT scans can calculate fatty infiltration with good reliability; however, they expose patients to high levels of radiation [43]. The point-of-care technology to examine the muscle quality at the early stage of developing muscle atrophy and fatty infiltration shows promise to implement preventive strategies before substantial irreversible rotator cuff injuries.

Rotator cuff disease is a prevalent musculoskeletal condition accounting for more than USD 3 billion in surgical costs alone [1,2,44]. Risk factors associated with rotator cuff disease are mostly unmodifiable, including age, sex, and genetics [45]. The presence of fatty infiltration is undesirable in patients undergoing rotator cuff repair. The biological mechanisms underpinning rotator cuff fatty infiltration remain poorly understood. However, what is clear is the progressive and predictable nature of fatty deposits within the rotator cuff muscles and the poor prognosis associated with higher concentrations of fat [46]. Despite this significant clinical importance of identification, current clinical standards use qualitative or semi-quantitative means to evaluate fatty infiltration. Therefore, a cost-effective and time-efficient quantitative imaging modality is needed to objectively measure fat fraction values accurately and reliably. The emergence of the Dixon sequencing in musculoskeletal imaging has the potential to fill this clinical gap; however, despite increasing awareness of its utility and further studies firmly validating not only the reliability and accuracy but also streamlining, post-processing remains a significant hurdle. Additionally, future work should focus on isolating whether changes in fat fraction have consequences beyond surgical planning. Phosphorus magnetic resonance imaging and blood oxygen-level-dependent (BOLD) imaging are two potential modalities uniquely positioned to pinpoint potential metabolic changes accompanied by the changes in rotator cuff fatty infiltration [47,48].

The present study has significant strength as the first systematic review of Dixon MRI methods for the quantification of rotator cuff fatty infiltration. Several limitations are associated with the utility of Dixon MRI sequencing, including being limited to application on modern equipment and requiring advanced image processing and reconstruction. Similarly, fat fraction was calculated from non-standardized algorithms, but its effectiveness and usability were not comparable across the included studies. The variance among studies both in sequencing parameters and post-processing limited our ability to pool data for further meta-analysis. To date, the use of Dixon sequencing for the calculation of fat fraction values of the rotator cuff remains in its infancy, with further robust studies validating the modality.

## 4. Materials and Methods

### 4.1. Search Strategy

A systematic literature review was undertaken to identify scientific articles utilizing a Dixon method magnetic resonance imaging (MRI) sequence for the quantitative evaluation of fatty infiltration within the rotator cuff muscles. The review adhered to the PRISMA guidelines [49]. The literature search was conducted on 27 February 2023 utilizing two databases: Ovid MEDLINE and PubMed. The search strategy involved a combination of keywords pertinent to the application of the Dixon method for quantifying rotator cuff fatty infiltration. Specifically, search terms included variations of “Dixon” or “modified Dixon” for Dixon MRI, “fatty infiltration” or “fatty degeneration” for fatty infiltration, and “rotator cuff” and “shoulder muscle” to target the rotator cuff muscles. A detailed list of search terms for each database is outlined in Appendix A.

### 4.2. Eligibility Criteria

The inclusion criteria were the following: (1) quantitative analysis of fatty infiltration by a Dixon MRI sequence; (2) quantitative analysis on at least one rotator cuff muscle; and (3) analysis conducted on human subjects. Studies were excluded if (1) the study population included evaluation of the rotator cuff in animals or cadavers; (2) the studies did not include at least one Dixon MRI sequence; or (3) the studies were written in a language other than English.

### 4.3. Data Extraction

The identified references were distributed to two independent reviewers (A.J.N. and J.H.), and abstracts were independently reviewed for inclusion. If there was disagreement about the inclusion of a study, a third reviewer (Y-S.L.) served as a tiebreaker. Finally, two authors (A.J.N. and J.H.) critically reviewed the full-text articles and summarized the findings. The following data were extracted for descriptive analysis and comparison of imaging sequences and post-image processing software used to quantify rotator cuff fatty infiltration from raw Dixon images.

### 4.4. Assessments of Study Quality and Risk of Bias

The quality of the studies was independently assessed by two review authors (A.J.N. and J.H.). If there was disagreement or discrepancies about the assessments, a third reviewer (Y-S.L.) was included in the discussion. The studies included in the analysis underwent systematic evaluation employing the GRADE framework, which assesses the quality of evidence [50]. Each study was evaluated across five categories: study population, imaging modality assessment, outcome evaluation, study design, and quantitative data analysis. Initially, all studies began with a “high” quality rating. Risk of bias assessment and applicability concerns were conducted in line with the Quality Assessment of Diagnostic Accuracy Studies-2 (QUADAS-2) [28]. The QUADAS-2 is subdivided into ‘Risk of Bias’ and ‘Applicability Concerns’ and is categorized as a low, high, or unclear risk or applicability concern. Within the risk of bias section, there are four domains, each representing foundational components of a diagnostic accuracy-based scientific paper: patient selection, index test, reference standard, and flow and timing. As for the applicability concerning subsection of the QUADAS-2, three domains are identified to gauge the external validity of the scientific paper and include the following: patient selection, index test, and reference standard. The detection of study outcomes demonstrating moderate or substantial effect sizes (i.e., indicated by a lower limit of 95% confidence interval, odds ratio > 2.0) may result in an elevation of the evidence quality. In total, all items across five categories for quality assessment were assessed: (1) study population, (2) assessment of Dixon MRI sequence, (3) assessment of post-image processing, (4) assessment of the reliability of Dixon sequence and post-image processing, and (5) study design. The risk of bias and applicability concern criteria were scored as “high”, “low”, or “unclear”.

## 5. Conclusions

The present systematic review suggests that fat fraction derived from multi-point Dixon imaging is an emerging and non-invasive method that provides an accurate, reliable, and quantitative measurement of rotator cuff fatty infiltration without the need for a tissue biopsy. Despite the variances in imaging sequencing and post-processing protocols that exist across the included studies, findings from the studies demonstrated that a difference in fat fraction was observed between people with various degrees of rotator cuff tear and those without any shoulder pathology. Thus, multi-point Dixon imaging has the potential to be utilized clinically, in lieu of the Goutallier classification scale, to objectively quantify fatty infiltration and shows promise in leading to improved clinical decision making for patients with rotator cuff tears.

## Figures and Tables

**Table 4:** GRADE ratings for the quality of articles based on the risk of bias, imprecision, inconsistency, indirectness, and publication bias.

Study	(1) Inconsistency	(2) Indirectness	(3) Imprecision	(4) Publication Bias	(5) Risk of Bias	Overall Quality Score (0–5)
Lee 2015 [19]	No	No	Yes	No	Yes	3
Nozaki 2015 [20]	No	No	Yes	No	No	4
Agten 2016 [21]	No	No	Yes	No	No	4
Nozaki 2016 [22]	No	No	Yes	No	No	4
Horiuchi 2017 [23]	No	No	Yes	No	No	4
Matsumura 2017 [12]	No	No	No	No	No	5
Kalin 2018 [13]	No	No	No	No	No	5
Yoon 2018 [24]	No	No	Yes	No	Yes	3
Khanna 2019 [14]	No	No	Yes	No	No	4
Kalin 2019 [25]	No	No	Yes	No	No	4
Hahn 2021 [15]	No	No	Yes	No	No	4
Xu 2023 [26]	No	No	No	No	No	5

**Table 5:** QUADAS-2 risk of bias and applicability concerns.

	Risk of Bias Rating of Included Articles				Applicability Concerns		
Study	Patient Selection	Index Test	Reference Standard	Flow and Timing	Patient Selection	Index Text	Reference Standard
Lee 2015 [19]	High	Low	Low	Low	Low	High	High
Nozaki 2015 [20]	High	Low	Low	High	Low	High	Low
Agten 2016 [21]	High	Low	Low	Low	High	Low	High
Nozaki 2016 [22]	High	Low	Low	Low	High	High	High
Horiuchi 2017 [23]	Low	Low	Low	Low	High	High	High
Matsumura 2017 [12]	High	Low	Low	Low	Low	Low	Low
Kalin 2018 [13]	Unclear	Low	Low	Low	High	Low	Low
Yoon 2018 [24]	Unclear	Low	Low	Low	High	High	High
Khanna 2019 [14]	Low	Low	Low	Low	High	High	High
Kalin 2019 [25]	Low	Low	Low	Low	High	Low	Low
Hahn 2021 [15]	Low	Low	Low	Low	High	High	Low
Xu 2023 [26]	High	Low	Low	Low	High	Low	High

**Table 6:** Summary of study results.

Study	Study Purpose	Stated Hypothesis	Results
Lee 2015 [19]	To assess usefulness of T2*-corrected FF map from VIBE MR Sequence	Not specified	T2*-corrected FF maps provide higher correlation (*r* = 0.78–0.95) than uncorrected FF maps (*r* = 0.59–0.72) from VIBE sequence. Excellent agreement of fat–water phantoms and quantitative T2*-corrected FF maps from IP and OP VIBE (*r* = 0.9096, *p* < 0.01).
Nozaki 2015 [20]	Quantify fatty degeneration of supraspinatus muscle using 2-point Dixon to evaluate correlation of atrophy and FF among different severities of rotator cuff tears	Not specified	FF values were greatest in the full-tear group (0.258 ± 0.123) followed by the partial tear group (0.166 ± 0.067) and no tear group (0.128 ± 0.061), *p* < 0.001. Low correlation between age and FF (*r* = 0.348) and high correlation between atrophy and FF values (*r* = 0.664).
Agten 2016 [21]	Analyze reliability of T2*-corrected multi-echo 3D Dixon to quantify specifically low-fat content of the supraspinatus	Not specified	Multi-echo 3D Dixon sequencing is able to reliably quantify low FF in the supraspinatus muscle with moderate concordance correlation coefficient (CCC = 0.641) to MR spectroscopy. Test–retest reliability was strong (rater 1 ICC = 0.757 and rater 2 ICC = 0.873). Inter-reader reliability for Dixon was strong (ICC = 0.893).
Nozaki 2016 [22]	Determine degree of pre-operative FI, longitudinal post-operative FI, and differences in FI of those who re-tear and those who do not	Pre-operative FF predicts re-tear Rotator cuff repair prevents progression of FI	Pre-operative supraspinatus and subscapularis FFs were significantly higher in the failed repair group (37.0% vs. 19.5%, *p* = 0.001 and 19.7% vs. 12.3%, *p* = 0.001, respectively) but not infraspinatus. Post-operative FFs of the subscapularis at 1-year follow-up were significantly higher in the intact repair group compared to normal tendon group (21.3% vs. 12.2%, *p* = 0.001).
Horiuchi 2017 [23]	Determine inter- and intra-rater reliability of 2-point Dixon and compare FF with Goutallier	FF will be more reliable and reproducible compared to Goutallier	Inter-observer agreement for quantitative evaluation of FFs among five readers was excellent (ICC = 0.893) with intra-rater coefficients being excellent for all readers (ICCs > 0.893). Intra-observer agreement of FF values for each reader ranged from 0.904 to 0.966.
Matsumura 2017 [12]	Assess FF and muscle volume of the whole rotator cuff muscles and clarify characteristics of FI and atrophy	Not specified	FF in control group was 15.1 ± 2.4% in the supraspinatus, 14.4 ± 2.6% in the infraspinatus, 16.0 ± 3.1% in the subscapularis, and 15.4 ± 5.0% in the teres minor. Supraspinatus tear group had greater supraspinatus FF (27.7 ± 11.4%, *p* = 0.02). Massive tear group had greater supraspinatus FF (44.0 ± 9.9%, *p* = 0.001) and infraspinatus FF (41.2 ± 9.8%, *p* < 0.001). Massive tear group only, fat-free muscle volume decreased in the supraspinatus (11.5 ± 4.1 cm^3^, *p* = 0.003) and in infraspinatus (28.9 ± 12.4cm^3^, *p* < 0.001), and increased in teres minor (18.0 ± 9.2cm^3^, *p* = 0.004).
Kalin 2018 [13]	Provide mean values for FF and muscle volume of bilateral rotator cuff and deltoid muscles in asymptomatic adults and investigate the influence of gender, age, and arm dominance	Not specified	Women had higher subscapularis FF values than men (7.5 ± 2.8% vs. 6.8 ± 1.9%, *p* = 0.001) but no other muscles. Men had higher muscle volume for all muscles (*p* < 0.001). The teres minor and deltoid muscles of the dominant arm had greater muscle volume for both genders (*p* < 0.009). FF values of the supraspinatus were lower in the dominant arm of men (*p* < 0.001). FF values of the shoulder muscles: supraspinatus 7.6 ± 2.7%, infraspinatus 5.7 ± 1.9%, subscapularis 7.2 ± 2.5%, teres minor 7.4 ± 2.7%, and deltoid 6.8 ± 3.1%.
Yoon 2018 [24]	Determine association between MRI findings of adhesive capsulitis and FF	Chronic disuse of shoulder muscles associated with adhesive capsulitis may be accompanied by FI	Supraspinatus FF was higher in those with extracapsular hyperintensity (3.00 ± 1.74% versus 1.81 ± 0.80%, *p* = 0.022). Inter-observer agreement was excellent for all shoulder muscles (ICCs: 0.955–0.992). FF values of the shoulder muscles: supraspinatus 2.5 ± 1.53%, infraspinatus 4.23 ± 2.45%, teres minor 3.41 ± 3.12%, subscapularis 3.49 ± 2.86%, teres major 4.49 ± 3.14%, and posterior deltoid 2.91 ± 1.85%.
Khanna 2019 [14]	Establish the reproducibility, minimal clinically important differences, and concurrent validity of 3D whole muscle volume and distribution of FI	Not specified	Good to excellent intra-rater reliability of sagittal divisions (ICC = 0.93–0.99) and axial divisions (ICC = 0.78–0.99). Very good to excellent inter-rater reliability for FF (ICC = 0.82–0.99) and volume (ICC = 0.92–0.99). Good to excellent agreement on concurrent validity with commercialized software (AnalyzeDirect 11.0) (ICC = 0.66–0.99).
Kalin 2019 [25]	Establish FF values of rotator cuff muscles and investigate age-associated changes between genders and muscles	Not specified	Inter-rater reliability for cross-sectional area on mDixon (ICC = 0.99) and FF values (ICC = 0.75) was excellent and good, respectively. Weak correlation between age and FF values of the rotator cuff muscles (*r* = 0.273, *p* < 0.05). Male FF values: supraspinatus 7.6 ± 1.5%, infraspinatus 6.4 ± 2.0%, teres minor 6.3 ± 2.0%, and subscapularis 6.4 ± 0.9%. Female FF values: supraspinatus 9.1 ± 2.4%, infraspinatus 6.6 ± 2.0%, teres minor 6.9 ± 1.5%, and subscapularis 7.0 ± 3.2%.
Hahn 2021 [15]	Compare diagnostic ability of mDixon TSE and evaluate feasibility of mDixon IP images to measure FF and size of rotator cuff muscles	Diagnostic ability of mDixon TSE would be comparable to conventional MRA and would accurately quantify FF and muscle size	Pre-operative agreement between fat-saturated T1W and mDixon TSE T1W water images in 176 of 183 tendons (96.2%) showed agreement in pre-operative diagnosis. Pre-operative agreement between reviewers in 155 of 183 tendons (84.7%). Post-operative agreement between fat-saturated T1W and mDixon water images in 55 of 57 tendons (96.5%). Post-operative agreement between reviewers in 52 of 57 tendons (91.2%). Strong linear correlation between FF values from mDixon and fat–water phantoms (*r* = 0.994). Excellent correlation of occupation ratio between mDixon and T1W (0.986).
Xu 2023 [26]	Determine associations between 3D overall FI and localized FI and assess feasibility of predicting overall FI with SAU	Some SAU-FIs will have high correlations with overall FI	Pearson and Spearman correlations between the supraspinatus overall FI and SAU-FIs were highest in the 2/3 (0.95 and 0.98), 3/6 (0.97 and 0.97), 4/6 (0.97 and 0.97), and 5/12 to 7/12 (0.95 to 0.96) SAU-FIs. The middle third of the supraspinatus is the best candidate location to predict overall FI.

FF, fat fraction; VIBE, volumetric interpolated breath-hold examination; FI, fatty infiltration; IP, in phase; OP, opposed phase; ICC, intra-class coefficient; CCC, concordance correlation coefficient; TSE, turbo spin echo; T1W, T1 weighted; SAU, sectional accumulation units.

## Data Availability

All data underlying this article will be shared upon reasonable request to the corresponding authors.

## Appendix A

The complete list of search terms used for the literature search are as follows: PubMed: (((dixon[Title/Abstract]) OR (modified dixon[Title/Abstract])AND (fatty infiltration[Title/Abstract]) OR (fatty degeneration[Title/Abstract]))) AND (rotator cuff[Title/Abstract]) OR (shoulder muscle[Title/Abstract]) and OVID Medline: ((dixon or modified dixon) and (fatty infiltration or fatty degeneration) and (rotator cuff or shoulder muscle)).
